# Crystal structure of a polyglycine hydrolase determined using a *RoseTTAFold* model

**DOI:** 10.1107/S2059798323000311

**Published:** 2023-02-06

**Authors:** Nicole V. Dowling, Todd A. Naumann, Neil P. J. Price, David R. Rose

**Affiliations:** a Department of Biology, University of Waterloo, 200 University Avenue West, Waterloo, ON N2L 3G1, Canada; b Mycotoxin Prevention and Applied Microbiology Research Unit, USDA, Agricultural Research Service, National Center for Agricultural Utilization Research, 1815 North University Street, Peoria, IL 61604, USA; c Renewable Product Technology Research Unit, USDA, Agricultural Research Service, National Center for Agricultural Utilization Research, 1815 North University Street, Peoria, IL 61604, USA; University of Cambridge, United Kingdom

**Keywords:** *RoseTTAFold*, fungi, chitinase-modifying proteins, polyglycine hydrolases, β-lactamases

## Abstract

Polyglycine hydrolases (PGHs) are secreted fungal proteases that cleave the polyglycine linker of *Zea mays* ChitA, a defensive chitinase, thus overcoming one mechanism of plant resistance to infection. Despite their importance in agriculture, there has been no previous structural characterization of this family of proteases. The objective of this research was to investigate the proteolytic mechanism and other characteristics by structural and biochemical means. Here, the first atomic structure of a polyglycine hydrolase was identified. It was solved by X-ray crystallography using a *RoseTTAFold* model, taking advantage of recent technical advances in structure prediction. PGHs are composed of two domains: the N- and C-domains. The N-domain is a novel tertiary fold with an as-yet unknown function that is found across all kingdoms of life. The C-domain shares structural similarities with class C β-lactamases, including a common catalytic nucleophilic serine. In addition to insights into the PGH family and its relationship to β-lactamases, the results demonstrate the power of complementing experimental structure determination with new computational techniques.

## Abstract 2

The atomic structure of a polyglycine hydrolase solved using a *RoseTTAFold* model-assisted molecular-replacement method reveals a two-domain enzyme. The structure reveals a novel tertiary fold and a similarity to β-lactamases.

## 1. Introduction

The phase problem has traditionally been a major bottleneck during structure solution by X-ray crystallography. In recent years, however, there has been a disruptive advance in available tools within structural biology. Previously, phases were either determined experimentally with multiple diffraction experiments or, more commonly, by molecular replacement of a highly similar experimental structure. Without experimental phases or an adequate structural model, researchers were forced to turn to protein modelling. Prior to the release of *RoseTTAFold* and *AlphaFold*, sequence-based protein modelling was quite limited (Baek *et al.*, 2021 ▸; Tunyasuvunakool *et al.*, 2021 ▸). Such modelling relied heavily on the sequence and structural similarity of experimentally determined structures. Recent advances in modelling methods have introduced a powerful new option for structural biologists. Novel protein structural analyses with limited similarity to current experimental structures are often no longer stalled by experimental phasing.

Polyglycine hydrolases are secreted fungal proteases that selectively cleave the polyglycine linker that connects the two functional domains of *Zea mays* chitinase ChitA. Their ability to cleave ChitA was first observed when protein extracts from corn ears rotted by the fungus *Cochliobolus carbonum* (syn. *Bipolaris zeicola*) were found to have altered chitinase activity profiles (Naumann *et al.*, 2009 ▸). Based on the observed activity, the altered chitinase was purified and identified as ChitA (Naumann *et al.*, 2009 ▸). The polyglycine-cleaving activity of the fungal protease, named Bz-cmp, was later described (Naumann *et al.*, 2014 ▸) and the identity of Bz-cmp was determined, facilitated by the development of next-generation sequencing technologies (Naumann *et al.*, 2015 ▸). Analysis of the primary structure of Bz-cmp shows that it consists of an amino-terminal domain with a novel sequence and a carboxy-terminal domain that resembles bacterial β-lactamases. Polyglycine hydrolases are part of a larger group of fungal proteases that separate the domains of ChitA and homologous chitinases called chitinase-modifying proteins (CMPs). Two other types of CMPs, fungalysin metallo­proteases (Naumann, 2011 ▸) and PA domain-containing subtilases named kilbournases (Naumann *et al.*, 2020 ▸), have been identified, but they do not cleave polyglycine targets.

To date, there are very few examples in nature that describe a polyglycine proteolytic target. In addition to *C. carbonum*, polyglycine hydrolase-encoding genes are present in the genomes of many fungi in the class Dothideomycetes. Es-cmp from *Epicoccum sorghi* is the most well characterized polyglycine hydrolase due to its high level of expression both in fungal cultures (Naumann *et al.*, 2014 ▸) and when expressed recombinantly in the yeast *Komagataella phaffii* (*syn. Pichia pastoris*) (Naumann *et al.*, 2015 ▸). Polyglycine hydrolase-encoding genes are also present in the genomes of some fungi of the related order Sordariomycetes, including *Fusarium vanettenii* (syn. *Fusarium solani* f. sp. *pisi*; syn. *Nectria haematococca*), a plant pathogen that does not infect corn (Coleman *et al.*, 2009 ▸). Interestingly, a few examples of polyglycine hydrolases are also present in the fungal division Basidiomycota, including the mushroom-producing wood-rot fungus *Galerina marginata* (Riley *et al.*, 2014 ▸). Despite preliminary biochemical characterization of Bz-cmp and Es-cmp (Naumann *et al.*, 2014 ▸), relatively little is known about these enzymes. The focus of our work is to investigate these novel proteases by structural and biochemical means in order to better understand their proteolytic mechanism and other characteristics.

In the present paper, we discuss the structure of one of these polyglycine hydrolases from *F. vanettenii*. The structure was solved by molecular replacement using a *RoseTTAFold* model (Baek *et al.*, 2021 ▸). The preliminary structure was determined using *MOLREP* and *Buccaneer* before being refined using *REFMAC* (Vagin & Teplyakov, 2010 ▸; Cowtan, 2006 ▸; Murshudov *et al.*, 2011 ▸). The structure solution depicts two distinct domains, referred to throughout as the N- and C-domains. The N-domain exhibits a previously structurally uncharacterized tertiary fold, with predicted fungal ties. Our analysis shows that this tertiary fold is the first to be reported in an experimentally determined structure. The C-domain resembles a fungal β-lactamase domain fold, although with proteolytic rather than β-lactamase activity.

## 2. Materials and methods

### 2.1. Cloning of expression plasmids and integration into *K. phaffii*

Cloning of the Fvan-cmp expression plasmid pTAN163 and integration of the linearized plasmid into the genome of *K. phaffii* to create expression strain TAN563 have been described previously (Naumann *et al.*, 2022 ▸). The Gm-cmp expression plasmid pTAN170 was cloned in a similar way and integrated into the *K. phaffii* genome to create expression strain TAN423. For cloning, genomic DNA was isolated from *G. marginata* CBS 339.88 and used as a PCR template, and the two exons of Galma1_254471 were amplified using oligo­nucleotides KS242 (GAGAGGCTGAAGCTGAATTCTCTCCCACTGACCTTTCTCTCAAAC) and KS243 (CCCCAGACCGCATGCGTATGAATGAAATTCGCCAG) for the first exon or KS244 (CATACGCATGCGGTCTGGGGAATAGGTCCTCGTCC) and KS245 (AGATGAGTTTTTGTTCTAGATCAAACAGTGGGATATGCATTCAAG) for the second exon. The expression plasmids pTAN259, pTAN260 and pTAN261 for the expression of Fvan-cmp(F543G), Fvan-cmp(R563K/D564T) and Fvan-cmp(F543G/R563K/D564T), respectively, were cloned using synthetic DNAs (Integrated DNA Technologies, Coralville, Iowa, USA) to create *K. phaffii* expression strains TAN617, TAN618 and TAN619.

### 2.2. Fvan-cmp purification

Recombinant Fvan-cmp protein was produced by heterologous strains of *K. phaffii* and was purified from expression cultures as described previously for Bz-cmp and Es-cmp (Naumann *et al.*, 2015 ▸).

### 2.3. Polyglycine hydrolase enzymatic activity

Fvan-cmp and Gm-cmp activity on corn ChitA was tested as detailed previously (Naumann *et al.*, 2015 ▸) by adding protease to solutions containing 1 m*M* ChitA in buffer (10 m*M* sodium acetate pH 5.2) followed by incubation at 30°C for 1 h prior to analysis by SDS–PAGE or matrix-assisted laser desorption/ionization time-of-flight mass spectrometry (MALDI-TOF MS). The N-terminal peptides released by the polyglycine hydrolase proteolytic activity were assayed by MALDI-TOF MS essentially as described previously (Naumann *et al.*, 2015 ▸). The instrument used was a Bruker Daltonics Microflex LRF (Bruker Daltonics, Billerica, Massachusetts, USA) with a pulsed N_2_ laser (337 Hz, 60 Hz pulse, 3000 shots) and with reflectron acquisition. The matrix used was 2,5-dihydro­benzoic acid (2,5-DHB). Mass analysis was performed using *Peptide Mass Calculator* v.3.2 (https://rna.rega.kuleuven.be/masspec/pepcalc.htm).

β-Lactamase activity was tested using the colorimetric substrate nitrocefin as described previously (O’Callaghan *et al.*, 1972 ▸). For purified Fvan-cmp, 200 n*M* enzyme was incubated with substrate for 24 h at 30°C. For mutants, cell-free medium was concentrated tenfold by ultrafiltration and added at 10% of the assay volume. No activity was observed.

### 2.4. Crystallization

Fvan-cmp protein was stored in 20 m*M* Tris–HCl pH 7.5. Crystals were obtained at 14°C by the hanging-drop vapour-diffusion method. The drops were set up using 1 µl reservoir solution and 1 µl Fvan-cmp at 21 mg ml^−1^ equilibrated against 500 µl reservoir solution. Fvan-cmp crystallized in the presence of 0.6 *M* sodium chloride, 0.1 *M* MES pH 6.5 and 20% PEG 4000. The protein crystallized in a thick plate morphology clustered from a single nucleation point after 2–3 weeks. Crystals were cryoprotected in 10% PEG 400 with sodium chloride, MES pH 6.5 and PEG 4000 at the previously indicated concentrations.

### 2.5. Data collection

Data were collected on the home-source diffractometer at the University of Waterloo using a Rigaku RUH3R rotating-anode generator and a Rigaku R-AXIS IV^++^ detector. Data collection took place at a temperature of 93 K and a wavelength of 1.54 Å. Diffraction data were processed with *Structure Studio* and *HKL*-2000 (Otwinowski & Minor, 1997 ▸). Fvan-cmp protein crystals diffracted to a resolution of 2.2 Å and appeared to belong to space group *P*2_1_2_1_2_1_. The asymmetric unit contained one molecule. There was no evidence of oligomerization in solution or in the crystal. Data-collection statistics are reported in Table 1 ▸.

### 2.6. *RoseTTAFold* model generation

The full sequence of Fvan-cmp was submitted to the *Robetta* server for model generation, only selecting the *RoseTTAFold* modelling method (Baek *et al.*, 2021 ▸). *Rose­TTAFold* is a fully automated process that combines *ab initio* modelling with comparative protein modelling. The output of the server gave five models of the structure. All models ranged from residues 13 to 616, with the first 12 residues remaining unmodelled. We chose to use the first model based on the metrics presented within the interface. The model was truncated by including coordinates with a predicted error of less than 3 Å.

### 2.7. Structure determination and refinement

Phases were not able to be obtained experimentally so molecular replacement was conducted on data for Fvan-cmp using the *RoseTTAFold* model (Baek *et al.*, 2021 ▸). Molecular replacement was performed in *MOLREP* within the *CCP*4 suite (Winn *et al.*, 2011 ▸; Vagin & Teplyakov, 2010 ▸; Murshudov *et al.*, 2011 ▸). The glycans were built using the carbohydrate module within *Coot* in *CCP*4 (Emsley & Cowtan, 2004 ▸; Emsley *et al.*, 2010 ▸). The Fvan-cmp structure was refined using successive rounds of *Privateer*, *REFMAC* and *Coot* (Agirre *et al.*, 2015 ▸; Murshudov *et al.*, 2011 ▸).

## 3. Results

### 3.1. Activity of polyglycine hydrolase homologs

**Figure 1:**
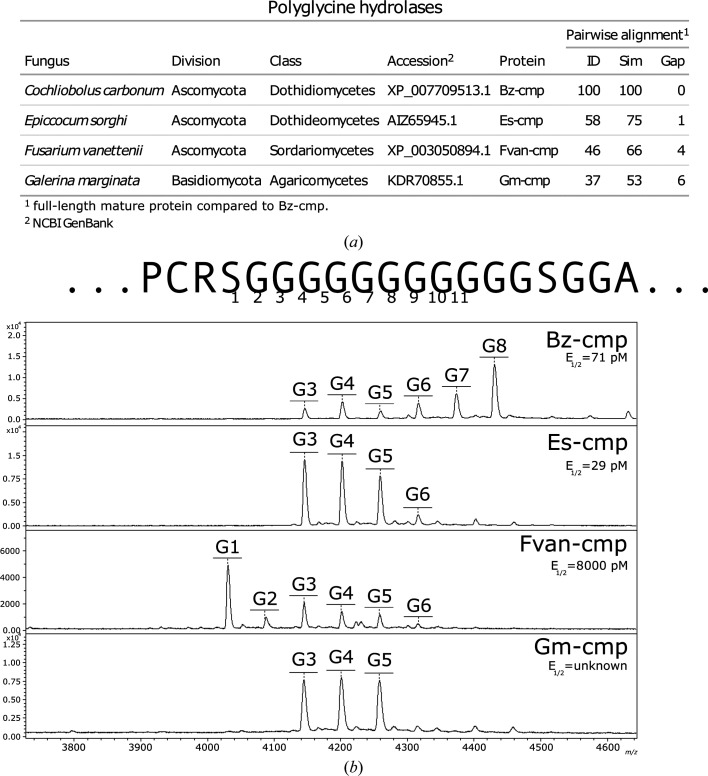
Polyglycine hydrolase homologs. (*a*) Comparison of primary structure. The sequence of each mature PGH was compared with that of Bz-cmp. The identity (ID), similarity (Sim) and gap percentages (GAP) are summarized. (*b*) Peptide-bond selectivity. Each PGH was incubated with ChitA, followed by MALDI-TOF MS analysis of the amino-terminal reaction products. All products resulted from the cleavage of Gly–Gly bonds in the ChitA linker. The sequence of the ChitA polyglycine linker, plus four additional amino acids on each side, is shown above.

Polyglycine hydrolase cleavage of corn ChitA has previously been demonstrated for Bz-cmp from *C. carbonum* and Es-cmp from *E. sorghi*, two corn pathogens of the fungal class Dothideomycetes (Naumann *et al.*, 2014 ▸, 2015 ▸). To determine whether homologous proteins encoded by more distantly related fungi would also cleave the ChitA polylinker, we chose two additional homologs and expressed them recombinantly. We chose Fvan-cmp from *F. vanettenii*, a plant pathogen in the class Sordariomyctes that does not infect corn, and Gm-cmp from *G. marginata*, a wood-rot fungus from the division Basidiomycota. The level of sequence similarity for each mature protease compared with Bz-cmp was determined (Fig. 1 ▸ *a*). As expected, proteins from more distantly related fungi had lower identity (ID), lower similarity (Sim) and more gaps (Gap).

Cell-free media from yeast liquid cultures expressing Fvan-cmp and Gm-cmp were observed to truncate ChitA by SDS–PAGE-based protease assays (not shown). Fvan-cmp accumulated in the media and was purified following the same procedure as used for Bz-cmp and Es-cmp (Naumann *et al.*, 2015 ▸). The amount of Fvan-cmp necessary to convert half of ChitA to the truncated form under standard conditions (*E*_1/2_) was determined to be 8000 p*M*, which is 112-fold and 276-fold greater than that reported for Bz-cmp and Es-cmp, respectively (Naumann *et al.*, 2015 ▸). Although activity was observed for Gm-cmp, the protease did not accumulate in the media to a level that could be observed by SDS–PAGE followed by Coomassie staining and we were not able to purify the protease or determine the *E*_1/2_.

To compare the peptide-bond selectivity of the different PGHs, we performed MALDI-TOF MS-based protease assays, which allow visualization of the smaller amino-terminal domain that is released from the larger enzymatic domain upon cleavage of the ChitA polyglycine linker (Fig. 1 ▸ *b*). For Bz-cmp, Es-cmp and Fvan-cmp, reactions were performed with purified proteins under standard conditions and at PGH concentrations matching their respective *E*_1/2_: 71, 29 and 8000 p*M*, respectively. For Gm-cmp, 1 µl of cell-free medium was added per 10 µl of reaction mixture, and the incubation time was increased from 1 h to 16 h. MALDI-TOF MS analysis of reaction products confirmed that both Fvan-cmp and Gm-cmp cleave Gly–Gly bonds in the ChitA polyglycine linker (Fig. 1 ▸ *b*). Fvan-cmp cleaves preferentially after Gly1, although products cleaved after Gly2, Gly3, Gly4, Gly5 and Gly6 were evident. This selectivity differs from that of both Bz-cmp and Es-cmp (Naumann *et al.*, 2015 ▸; Fig. 1 ▸ *b*). Gm-cmp cleaved three different peptide bonds with similar frequency after Gly3, Gly4 and Gly5, similar to the selectivity of Es-cmp.

### 3.2. Fvan-cmp structure

**Figure 2:**
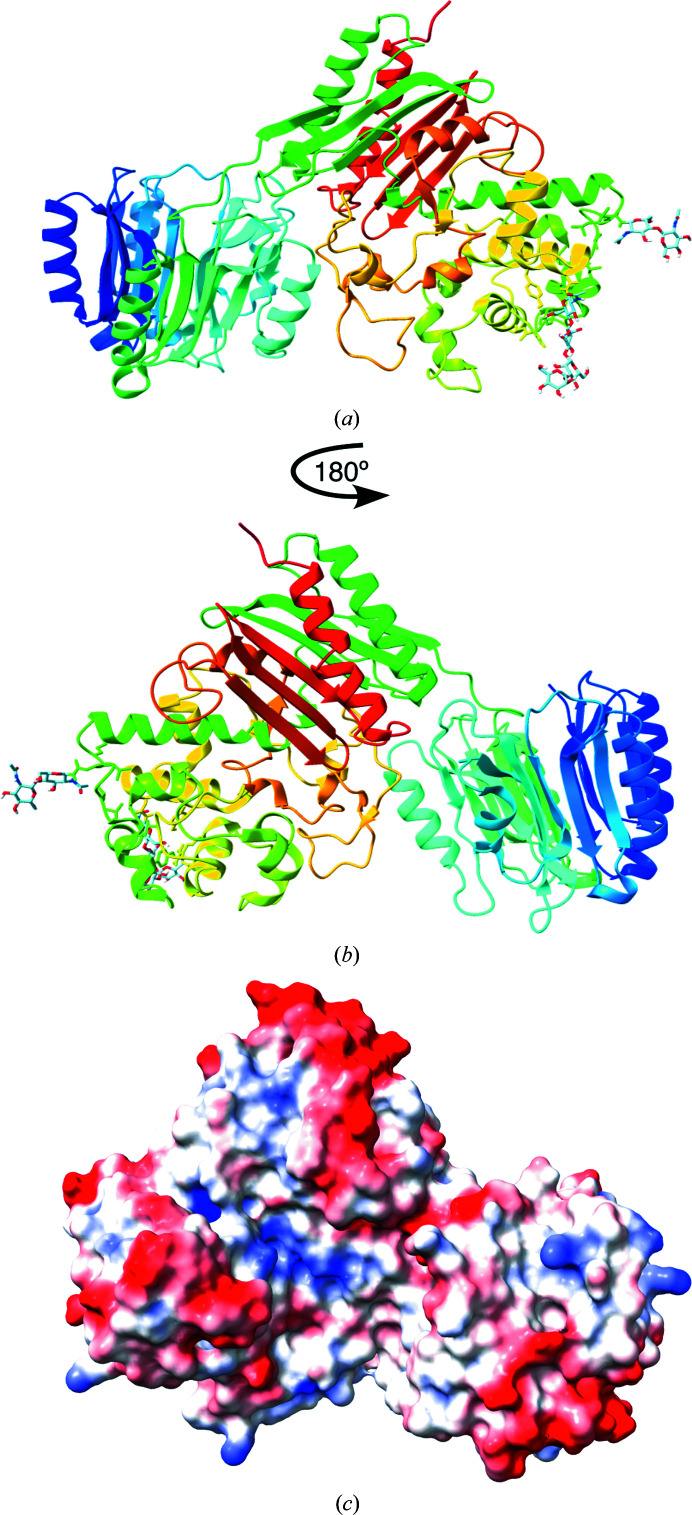
(*a*) Fvan-cmp structure with the N-domain on the left and the C-domain on the right. (*b*) Fvan-cmp structure rotated 180° from (*a*), which is the ideal orientation to view the active site of the structure. The N-glycosylation sites are represented as sticks on the C-domain. (*c*) The electrostatic potential surface map for Fvan-cmp at pH 5.0 in the orientation shown in (*b*). The active-site cleft is shown in blue (positive potential).

Of the polyglycine hydrolases discussed above, only Fvan-cmp produced crystals that were suitable for analysis. The structure of Fvan-cmp was solved to 2.19 Å resolution (PDB entry 7tpu) by molecular replacement of a *RoseTTAFold*-generated model, as discussed below. Fig. 2 ▸ illustrates the overall structure of the protein, representing 603 of the 616 amino-acid residues in the sequence and two glycosylation sites. The first 12 residues were omitted due to a lack of electron density present in the 2*F*_o_ − *F*_c_ and *F*_o_ − *F*_c_ maps. Fvan-cmp consists of two distinct domains, the N- and C-domains, that are connected by a linker. These domains will be discussed independently in the following sections.

### 3.3. N-domain

**Figure 3:**
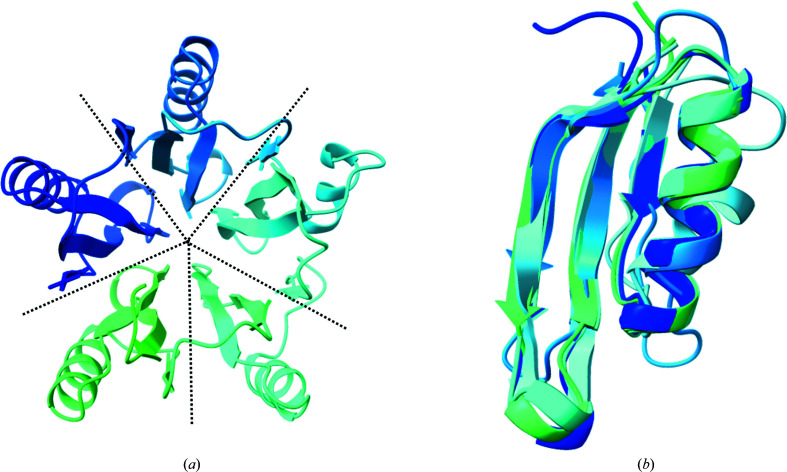
(*a*) Fvan-cmp structure in a top-down view of the N-domain. The five quasi-identical structural repeats that compose this domain are segregated visually by dotted lines. (*b*) A structural superposition of the repeats aligned by their C^α^ atoms.

The Fvan-cmp N-domain (residues 13–262) consists of four loops, five α-helices and 15 β-strands assembled into a distinct tertiary fold. This distinct structure, as shown in Fig. 3 ▸, is comprised of five quasi-identical structural repeats (Fig. 3 ▸ *b*) consisting of three β-sheets and an α-helix arranged as EHEE with β-strands in an antiparallel assembly. Each repeat spans 44 amino-acid residues with a 5–6-residue loop connecting them, as shown in Supplementary Fig. S2. These repeats are defined as structural repeats as there appears to be limited sequence conservation between the regions. When in the tertiary structure, these five regions arrange into a barrel-like structure.

When the structure was first solved, we found that the tertiary structure did not coincide with any known αβ-barrel folds but was identified as a novel superfamily in an analysis of the AlphaFold Protein Stucture Database (Bordin *et al.*, 2022 ▸). To investigate this, we conducted a search with two web servers: the *DALI* protein structure-comparison server and *FoldSeek* (Kempen *et al.*, 2022 ▸; Holm & Rosenström, 2010 ▸). Within the *DALI* search, we evaluated tertiary-fold likeness by the assigned *Z*-score metric. The *Z*-score is the similarity score between the query structure and its matches; strong matches have *Z*-scores higher than the assigned cutoff (Holm *et al.*, 2008 ▸). The *Z*-score cutoff is calculated based on the number of residues for the input query (Holm *et al.*, 2008 ▸). For the N-domain, the assigned *Z*-score cutoff was 24 and the closest match within the server had a *Z*-score of 4.5. Further investigation of the top hits revealed that there was no full match for the structural repeat or for the tertiary structure described. We ran a search using the *FoldSeek* web server against all currently available databases and found a similar but interesting result. As with *DALI*, there was no experimentally determined structure resembling the N-domain tertiary fold. However, *FoldSeek* did identify similar predicted structures within the AlphaFold Protein Structure Database. To date, none of these identified proteins has been functionally characterized.

The novelty of the N-domain explains the difficulties during the structure-solution process. The sequence search within the Protein Data Bank (https://www.rcsb.org/) did not identify an adequate model for molecular replacement (Berman *et al.*, 2000 ▸). Traditional automated modelling servers all failed to generate a full-length model. The partial coverage models failed in the molecular-replacement pipeline.

Recently, with the release of *RoseTTAFold* from the Baker laboratory, we were able to obtain a full-length sequence model owing to the sophistication of the deep-learning processing in *RoseTTAFold* (Baek *et al.*, 2021 ▸). The *Robetta* server (https://robetta.bakerlab.org) outputs the top five models from the run. Observing the per-residue error plot, we trimmed our model coordinates to those residues with a predicted error of less than 3 Å. We used the trimmed *RoseTTAFold* model to solve the structure by molecular replacement.

The accuracy of the secondary structures within the Fvan-cmp structure from *RoseTTAFold* is remarkable. A simple backbone alignment of the error-truncated *RoseTTAFold* model and the final structure had a final r.m.s.d. of 2.76 Å. This method is not reliable for determining side-chain orientations, nor is it capable of determining post-translational modifications, but it can be used as a powerful tool in conjunction with experimental data.

### 3.4. C-domain

**Figure 4:**
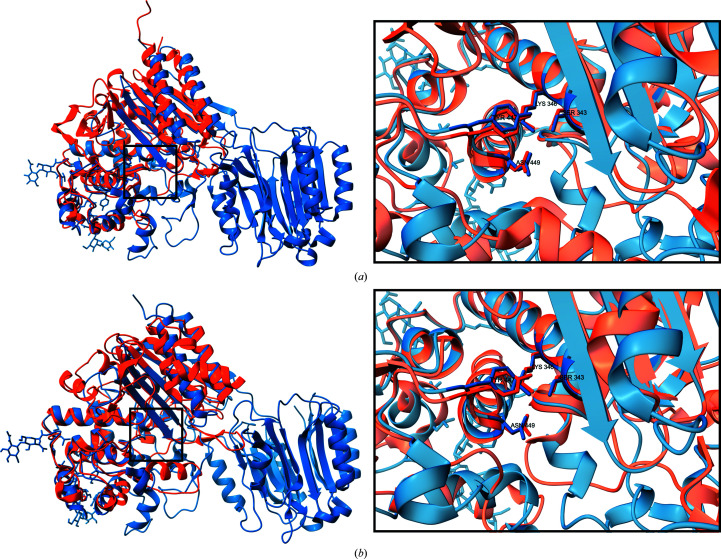
Structural alignment of a penicillin-binding protein (PDB entry 2qmi), a β-lactamase (PDB entry 4gzb) and a polyglycine hydrolase, Fvan-cmp (PDB entry 7tpu). The global structural alignment of PDB entries 7tpu and 2qmi (*a*) and 4gzb (*b*) is shown. For each alignment, a focused view shows the active-site secondary structures and their global arrangement. Residues 276–297 and 365–447 were omitted for better visualization of the active site. The two conserved β-lactamase motifs within Fvan-cmp are labelled and shown in stick representation: Ser343, Lys346, Tyr447 and Asn449. The corresponding residues are represented in stick form for the penicillin-binding protein and β-lactamase.

The Fvan-cmp C-domain (residues 271–616) consists of seven α-helices and one antiparallel β-sheet and resembles a β-lactamase fold. A *DALI* search against all structures within the Protein Data Bank yielded high *Z*-scores with penicillin-binding proteins and β-lactamases. Structural alignment of the Fvan-cmp C-domain against a penicillin-binding protein (PDB entry 2qmi) and a β-lactamase (PDB entry 4gzb) showed a strong similarity to the β-lactamase fold (Delfosse *et al.*, 2009 ▸; Lahiri *et al.*, 2013 ▸). In Fig. 4 ▸, we show structural alignments of the β-lactamase domains of the three proteins: Fvan-cmp, 2qmi (Fig. 4 ▸ *a*) and 4gzb (Fig. 4 ▸ *b*). The αβα folds are conserved in global positioning between the three proteins.

Within the β-lactamase fold, three conserved sequence motifs are observed within penicillin-binding proteins and multiple classes of β-lactamases. Two of the three sequences (Table 2 ▸) are observed in Fvan-cmp, aligning with class C β-lactamases. β-Lactamases inactivate β-lactam antibiotics such as penicillins, cephalosporins and carbapenems, rendering them inactive, and are an important mechanism of bacterial antibiotic resistance. The class C β-lactamases are found solely in Gram-negative bacteria and the mechanism by which they hydrolyze β-lactam antibiotics is still incompletely understood (Page, 2020 ▸).

These motifs have previously been determined to play a prominent role in substrate orientation and catalysis in β-lactamases (Goldberg *et al.*, 2003 ▸). The first motif contains the nucleophile used within the observed enzymatic catalysis. Fvan-cmp shares the same nucleophilic serine found within this motif. The second and third motifs are involved in substrate positioning in penicillin-binding proteins (Sauvage *et al.*, 2008 ▸). Within the third motif, the glycine in the third position is important in preventing steric interference during substrate binding (Sauvage *et al.*, 2008 ▸). Fvan-cmp lacks this glycine and instead contains a phenylalanine in the same structural position. Fig. 4 ▸ shows a comparison of the active sites of Fvan-cmp to a reference penicillin-binding protein (PDB entry 2qmi) and a class C β-lactamase (PDB entry 4gzb). Despite the structural similarities between the chitinase-modifying proteins and penicillin-binding proteins/β-lactamases there are critical differences that have a large effect on enzymatic function.

### 3.5. β-Lactamase activity

As discussed previously, Fvan-cmp contains two of the three conserved sequence motifs found within penicillin-binding proteins and β-lactamases. Noting this, previous work tested two different polyglycine hydrolases, Bz-cmp and Es-cmp, for β-lactam binding and β-lactamase activity (Naumann *et al.*, 2015 ▸). The potential β-lactamase activity was tested on nitrocefin, a colorimetric substrate, but neither showed activity. The β-lactamase inhibitor clavulanic acid was also added to protease reactions containing Bz-cmp or Es-cmp, but inhibition of proteolysis of ChitA was not observed.

As Es-cmp did not exhibit β-lactam binding or β-lactamase activity and in view of its structural similarity to Fvan-cmp, we attempted to introduce β-lactamase activity through site-directed mutagenesis. Specifically, we reduced the proposed steric hindrance to the active site of Fvan-cmp using a single mutant (F534G) and restored the third conserved sequence motif using a double mutant (R563K/D564T). A triple mutant was also constructed (F534G/R563K/D564T). Expression of the mutants was greatly reduced compared with wild-type Fvan-cmp, as noted by SDS–PAGE analysis of cell-free media after induction (Supplementary Fig. S2). Despite the low level of protein that accumulated, purification of the single and double mutants was attempted, but resulted in loss of protein, indicating that they are likely to be misfolded. Utilizing a nitrocefin assay, we did not observe β-lactamase activity for the cell-free media of either Fvan-cmp or the single, double or triple mutants. Purified Fvan-cmp also lacked β-lactamase activity, as reported for Bz-cmp and Es-cmp.

## 4. Discussion

### 4.1. Novel N-domain tertiary fold

In our *FoldSeek* search we came across several predicted proteins within the AlphaFold Protein Structure Database that shared the tertiary fold of the N-terminal domain. All of these proteins exhibit the lack of sequence conservation between the individual structural repeats that we observed in Fvan-cmp. The proteins (an abbreviated list is given in Supplementary Table S1) are diverse in origin, spanning across all kingdoms, with the majority found in bacteria. These proteins vary in the level of functional characterization; however, they share a lack of functional descriptors for the tertiary fold described. In the literature, there is speculation that this N-domain might play a role in substrate positioning and/or exo-site binding of ChitA and ChitB (Naumann *et al.*, 2015 ▸, 2017 ▸). The level of conservation of this domain in all kingdoms suggests a more general function that is not specific to polyglycine hydrolases. The potential for this domain to be involved in protein–protein interactions (PPIs) is possible due to the structural repetitions as seen in other PPI domains (Andrade *et al.*, 2001 ▸; Schapira *et al.*, 2017 ▸; Freilich *et al.*, 2018 ▸). Examples might include a chaperone activity involved in the folding or stability of the rest of the protein, or a role in transporting or anchoring to ensure localization of the protein to a specific target. Our result opens up an area of future work, which will focus on determining the biological function of this tertiary fold and its importance across the kingdoms.

### 4.2. Polyglycine hydrolases and their relationship to β-lactamases

This paper has highlighted the similarities between the representative polyglycine hydrolase (Fvan-cmp), penicillin-binding proteins and class C β-lactamases. We showed that Fvan-cmp retains two of the three conserved β-lactamase motifs and the core active-site αβα fold but lacks the associated activity. It is reasonable to suggest that polyglycine hydrolases share a common ancestor protein with β-lactam­ases, as do β-lactamases and penicillin-binding proteins. Fungal lactamases have previously been described in the literature but lack the extensive characterization afforded to bacterial β-lactamases (Gao *et al.*, 2017 ▸).

Focusing on the residue similarities between β-lactamases and polyglycine hydrolases, we observed two important features. Firstly, in addition to the retained catalytic motifs, PGHs contain an analog of Tyr150 in AmpC β-lactamase (Tyr447 in Fvan-cmp). This residue makes an important distinction between the different classes of β-lactamases and is integral to the kinetic functioning of β-lactamases (Dubus *et al.*, 1994 ▸). Secondly, polyglycine hydrolases share conserved residues with other classes of β-lactamases. A recent study of class A β-lactamases categorized the conserved residues into ‘shells’. These shells can be defined by proximity to the active site and function (Chikunova & Ubbink, 2022 ▸). The conserved residues are implicated in the folding, stability and function of the protein. We found that the polyglycine hydrolases retained several of these residues (Supplementary Table S2).

The point mutagenesis and structural studies demonstrate that if the protein is properly folded in the cell-free media, the absence of β-lactamase activity could be due to regions outside the catalytic centre (refer to Supplementary Fig. S2). The Fvan-cmp active site and surface map (data not shown) depicts a region that is sterically limited. It may be that the flexibility of the polyglycine peptides requires that they be constricted into a narrow binding region in these hydrolases, a region that is incompatible with a bulkier lactam ring.

### 4.3. Application of new tools in structural science

*RoseTTAFold* and *AlphaFold* have provided new approaches to the field of structural biology (Baek *et al.*, 2021 ▸; Tunyasuvunakool *et al.*, 2021 ▸). Prior to these methods, sequence-based structure predictions were not accurate in the absence of experimental templates. The accuracy of predications has improved using *RoseTTAFold* and/or *AlphaFold* with accompanying searches for structurally similar proteins using the *DALI* server or *FoldSeek* (Holm & Rosenström, 2010 ▸; Holm & Laakso, 2016 ▸; Kempen *et al.*, 2022 ▸).

The work described here demonstrates both the power and limitations of these new tools. While the pipeline was critical to the structure determination of Fvan-cmp, there are still questions about the differing specificity and activity of the fungal polyglycine hydrolases as well as details of the catalytic mechanism that can only be addressed through experimentation. Nevertheless, the insights gained, and the hypotheses formed by these results, are an exciting advance for this family of proteins.

## Supplementary Material

PDB reference: Fvan-cmp, 7tpu

Supplementary Figures and Tables. DOI: 10.1107/S2059798323000311/rr5227sup1.pdf

## Figures and Tables

**Table 1:** Data-collection, refinement and validation statistics for Fvan-cmp (PDB entry 7tpu)

Data-collection statistics	
Wavelength (Å)	1.54178
Space group	*P*2_1_2_1_2_1_
*a*, *b*, *c* (Å)	80.80, 94.65, 110.48
α, β, γ (°)	90.00, 90.00, 90.00
Resolution range (Å)	53.76–2.19
Completeness (%)	98.9
Mean *I*/σ(*I*)	1.94 (at 2.20 Å)
Wilson *B* factor (Å^2^)	31.7
Refinement statistics	
*R*_work_/*R*_free_	0.197/0.254
Average *B* factor (Å^2^)	36.0
No. of protein atoms	9276
R.m.s.d., bond lengths (°)	0.008
R.m.s.d., angles (Å)	1.479
Validation statistics	
Ramachandran favoured (%)	100
Ramachandran outliers (%)	0
Clashscore	4

**Table 2:** Conserved sequence motifs in penicillin-binding proteins and β-lactamases The conserved sequence motifs found within the αβα β-lactamase fold occur in penicillin-binding proteins and multiple classes of β-lactamases. The motifs occur in different secondary structures in the same relative positions across different proteins. Fvan-cmp shares two of the three conserved motifs but lacks the third motif. The residues are identified by their sequence for clarity.

Conserved sequence		Location within fold	Fvan-cmp sequence
1	S-*X*-*X*-K	α-Helix	S_343_-V-S-K_346_
2	Y/S-X-N†	Active site facing loop, before α-helix	Y_447_-S-N_449_
3	K-T-G	Terminal β-strand on β-sheet	n/a

† Class A β-lactamases and penicillin-binding proteins contain a serine while class C β-­lactamases contain a tyrosine at the first position of this motif.

## References

[bb1] Agirre, J., Iglesias-Fernández, J., Rovira, C., Davies, G. J., Wilson, K. S. & Cowtan, K. D. (2015). *Nat. Struct. Mol. Biol.* **22**, 833–834. 10.1038/nsmb.3115 26581513

[bb2] Andrade, M. A., Perez-Iratxeta, C. & Ponting, C. P. (2001). *J. Struct. Biol.* **134**, 117–131. 10.1006/jsbi.2001.4392 11551174

[bb3] Baek, M., DiMaio, F., Anishchenko, I., Dauparas, J., Ovchinnikov, S., Lee, G. R., Wang, J., Cong, Q., Kinch, L. N., Schaeffer, R. D., Millán, C., Park, H., Adams, C., Glassman, C. R., DeGiovanni, A., Pereira, J. H., Rodrigues, A. V., van Dijk, A. A., Ebrecht, A. C., Opperman, D. J., Sagmeister, T., Buhlheller, C., Pavkov-Keller, T., Rathina­swamy, M. K., Dalwadi, U., Yip, C. K., Burke, J. E., Garcia, K. C., Grishin, N. V., Adams, P. D., Read, R. J. & Baker, D. (2021). *Science*, **373**, 871–876.

[bb4] Berman, H. M., Westbrook, J., Feng, Z., Gilliland, G., Bhat, T. N., Weissig, H., Shindyalov, I. N. & Bourne, P. E. (2000). *Nucleic Acids Res.* **28**, 235–242. 10.1093/nar/28.1.235 PMC102472 10592235

[bb5] Bordin, N., Sillitoe, I., Nallapareddy, V., Rauer, C., Lam, S. D., Waman, V. P., Sen, N., Heinzinger, M., Littmann, M., Kim, S., Velankar, S., Steinegger, M., Rost, B. & Orengo, C. (2022). *bioRxiv*, 2022.06.02.494367.

[bb6] Chikunova, A. & Ubbink, M. (2022). *Protein Sci.* **31**, e4328. 10.1002/pro.4328 PMC9112487 35634774

[bb7] Coleman, J. J., Rounsley, S. D., Rodriguez-Carres, M., Kuo, A., Wasmann, C. C., Grimwood, J., Schmutz, J., Taga, M., White, G. J., Zhou, S., Schwartz, D. C., Freitag, M., Ma, L., Danchin, E. G. J., Henrissat, B., Coutinho, P. M., Nelson, D. R., Straney, D., Napoli, C. A., Barker, B. M., Gribskov, M., Rep, M., Kroken, S., Molnár, I., Rensing, C., Kennell, J. C., Zamora, J., Farman, M. L., Selker, E. U., Salamov, A., Shapiro, H., Pangilinan, J., Lindquist, E., Lamers, C., Grigoriev, I. V., Geiser, D. M., Covert, S. F., Temporini, E. & VanEtten, H. D. (2009). *PLoS Genet.* **5**, e1000618. 10.1371/journal.pgen.1000618 PMC2725324 19714214

[bb9] Cowtan, K. (2006). *Acta Cryst.* D**62**, 1002–1011. 10.1107/S0907444906022116 16929101

[bb10] Delfosse, V., Girard, E., Birck, C., Delmarcelle, M., Delarue, M., Poch, O., Schultz, P. & Mayer, C. (2009). *PLoS One*, **4**, e4712. 10.1371/journal.pone.0004712 PMC2651629 19266066

[bb11] Dubus, A., Normark, S., Kania, M. & Page, M. G. P. (1994). *Biochemistry*, **33**, 8577–8586. 10.1021/bi00194a024 8031792

[bb12] Emsley, P. & Cowtan, K. (2004). *Acta Cryst.* D**60**, 2126–2132. 10.1107/S0907444904019158 15572765

[bb13] Emsley, P., Lohkamp, B., Scott, W. G. & Cowtan, K. (2010). *Acta Cryst.* D**66**, 486–501. 10.1107/S0907444910007493 PMC2852313 20383002

[bb14] Freilich, R., Arhar, T., Abrams, J. L. & Gestwicki, J. E. (2018). *Acc. Chem. Res.* **51**, 940–949. 10.1021/acs.accounts.8b00036 PMC6082625 29613769

[bb15] Gao, M., Glenn, A. E., Blacutt, A. A. & Gold, S. E. (2017). *Front. Microbiol.* **8**, 1775. 10.3389/fmicb.2017.01775 PMC5610705 28974947

[bb16] Goldberg, S. D., Iannuccilli, W., Nguyen, T., Ju, J. & Cornish, V. W. (2003). *Protein Sci.* **12**, 1633–1645. 10.1110/ps.0302903 PMC2323950 12876313

[bb17] Holm, L., Kääriäinen, S., Rosenström, P. & Schenkel, A. (2008). *Bioinformatics*, **24**, 2780–2781. 10.1093/bioinformatics/btn507 PMC2639270 18818215

[bb18] Holm, L. & Laakso, L. M. (2016). *Nucleic Acids Res.* **44**, W351–W355. 10.1093/nar/gkw357 PMC4987910 27131377

[bb19] Holm, L. & Rosenström, P. (2010). *Nucleic Acids Res.* **38**, W545–W549. 10.1093/nar/gkq366 PMC2896194 20457744

[bb20] Kempen, M. van, Kim, S. S., Tumescheit, C., Mirdita, M., Gilchrist, C. L. M., Söding, J. & Steinegger, M. (2022). *bioRxiv*, 2022.02.07.479398.

[bb21] Lahiri, S. D., Mangani, S., Durand-Reville, T., Benvenuti, M., De Luca, F., Sanyal, G. & Docquier, J.-D. (2013). *Antimicrob. Agents Chemother.* **57**, 2496–2505. 10.1128/AAC.02247-12 PMC3716117 23439634

[bb22] Murshudov, G. N., Skubák, P., Lebedev, A. A., Pannu, N. S., Steiner, R. A., Nicholls, R. A., Winn, M. D., Long, F. & Vagin, A. A. (2011). *Acta Cryst.* D**67**, 355–367. 10.1107/S0907444911001314 PMC3069751 21460454

[bb23] Naumann, T. A. (2011). *Mol. Plant Pathol.* **12**, 365–372. 10.1111/j.1364-3703.2010.00677.x PMC6640348 21453431

[bb24] Naumann, T. A., Bakota, E. L. & Price, N. P. J. (2017). *Protein Sci.* **26**, 1214–1223. 10.1002/pro.3175 PMC6383549 28383143

[bb25] Naumann, T. A., Naldrett, M. J. & Price, N. P. J. (2020). *Fungal Genet. Biol.* **141**, 103399. 10.1016/j.fgb.2020.103399 32387407

[bb26] Naumann, T. A., Naldrett, M. J., Ward, T. J. & Price, N. P. J. (2015). *Protein Sci.* **24**, 1147–1157. 10.1002/pro.2705 PMC4500313 25966977

[bb27] Naumann, T. A., Sollenberger, K. G. & Hao, G. (2022). *Protein Expr. Purif.* **194**, 106076. 10.1016/j.pep.2022.106076 35240278

[bb28] Naumann, T. A., Wicklow, D. T. & Kendra, D. F. (2009). *Physiol. Mol. Plant Pathol.* **74**, 134–141.

[bb29] Naumann, T. A., Wicklow, D. T. & Price, N. P. J. (2014). *Biochem. J.* **460**, 187–198. 10.1042/BJ20140268 24627966

[bb30] O’Callaghan, C. H., Morris, A., Kirby, S. M. & Shingler, A. H. (1972). *Antimicrob. Agents Chemother.* **1**, 283–288. 10.1128/aac.1.4.283 PMC444209 4208895

[bb31] Otwinowski, Z. & Minor, W. (1997). *Methods Enzymol.* **276**, 307–326. 10.1016/S0076-6879(97)76066-X 27754618

[bb32] Page, M. G. P. (2020). *Antimicrob. Agents Chemother.* **64**, e02247-19.

[bb33] Riley, R., Salamov, A. A., Brown, D. W., Nagy, L. G., Floudas, D., Held, B. W., Levasseur, A., Lombard, V., Morin, E., Otillar, R., Lindquist, E. A., Sun, H., LaButti, K. M., Schmutz, J., Jabbour, D., Luo, H., Baker, S. E., Pisabarro, A. G., Walton, J. D., Blanchette, R. A., Henrissat, B., Martin, F., Cullen, D., Hibbett, D. S. & Grigoriev, I. V. (2014). *Proc. Natl Acad. Sci. USA*, **111**, 9923–9928. 10.1073/pnas.1400592111 PMC4103376 24958869

[bb34] Sauvage, E., Kerff, F., Terrak, M., Ayala, J. A. & Charlier, P. (2008). *FEMS Microbiol. Rev.* **32**, 234–258. 10.1111/j.1574-6976.2008.00105.x 18266856

[bb35] Schapira, M., Tyers, M., Torrent, M. & Arrowsmith, C. H. (2017). *Nat. Rev. Drug Discov.* **16**, 773–786. 10.1038/nrd.2017.179 PMC5975957 29026209

[bb36] Tunyasuvunakool, K., Adler, J., Wu, Z., Green, T., Zielinski, M., Žídek, A., Bridgland, A., Cowie, A., Meyer, C., Laydon, A., Velankar, S., Kleywegt, G. J., Bateman, A., Evans, R., Pritzel, A., Figurnov, M., Ronneberger, O., Bates, R., Kohl, S. A. A., Potapenko, A., Ballard, A. J., Romera-Paredes, B., Nikolov, S., Jain, R., Clancy, E., Reiman, D., Petersen, S., Senior, A. W., Kavukcuoglu, K., Birney, E., Kohli, P., Jumper, J. & Hassabis, D. (2021). *Nature*, **596**, 590–596.

[bb37] Vagin, A. & Teplyakov, A. (2010). *Acta Cryst.* D**66**, 22–25. 10.1107/S0907444909042589 20057045

[bb8] Winn, M. D., Ballard, C. C., Cowtan, K. D., Dodson, E. J., Emsley, P., Evans, P. R., Keegan, R. M., Krissinel, E. B., Leslie, A. G. W., McCoy, A., McNicholas, S. J., Murshudov, G. N., Pannu, N. S., Potterton, E. A., Powell, H. R., Read, R. J., Vagin, A. & Wilson, K. S. (2011). *Acta Cryst.* D**67**, 235–242. 10.1107/S0907444910045749 PMC3069738 21460441

