# Supplementary material for: Crystal structure of a polyglycine hydrolase determined using a RoseTTAFold model
Source: Acta Crystallogr D Struct Biol. 2023 Feb 6;79(Pt 2):168–76. doi: 10.1107/S2059798323000311 (PMC9912923; doi:10.1107/S2059798323000311)
Supplement: Supplementary file 1 [file d-79-00168-sup1.pdf]

STRUCTURAL BIOLOGY

Volume 79 (2023)

Supporting information for article:

Crystal structure of a polyglycine hydrolase determined using a *RoseTTAFold* model

Nicole V. Dowling, Todd A. Naumann, Neil P. J. Price and David R. Rose

```
Bz-cmp      SPTTNQNLPSSKR-DATVEQN------VFFDINGKEVETRIAKLKAEGYRPTSLNIHGTP
Es-cmp      FPANQQDLTFAKR-NGTFEQS------VFYGLTGPEVEAKLAKLKADGYRPTSLNIHGST
Fvan-cmp    -------LPNQRRSNVTSHVE------TYYSVDGATHAEKSKALKADGYRIVSLSSYGSP
Gm_CMP      -SPTDLSLKRTVPSNTTLSSRSIPAWQFYYGVTGAQHQTSFNQWSVSGYRMISLSAYGQP
             *     : *        :: : *          ***  **  :*

Bz-cmp      TDAKYAGIWIKQDGNPYETIVGANKTTYDAWLDQWKASGYVSTQVSATGPASNAVFAGVM
Es-cmp      SDAKYAGIWTKQTGDDFETILGANKTVYDAWLDSHKAQGYVSTHVSATGGSSDALFAGVM
Fvan-cmp    DSANYAAIWVQEEGPSFEIIHDADEATYNSWLQTWKSRGYVSTQVSATGPAENAVFAGVM
Gm-CMP      PNHRYAAVWVQRSGPAYFAIHEASGSQYQTFFDSHTPNGYVSTVITVTGPANAAVYAGVM
           ** :* :  *  :  *  *  : *:::::    ***** :: ** :  *::****

Bz-cmp      EKIPSIRNWTQICGTSSP-YAYLNATGDAP-IVITGVSLYGTPTERQYCVLGHEDTDNHQ
Es-cmp      EKVPSVANWIQVCGLDNP-YAYANATIDEP-MYIKGVSMYGAPNERQYCILGHENLVNYQ
Fvan-cmp    ENIN-VANWFQSCELENP-WAFSNTTGNVD-VVVKGFRMFGTPEERRYCILGHENVGNEQ
Gm-CMP      EQNG-VTNWYQKCGLTNAQYATELNNGFANRYFLKSFTEYGTSSDRRFCGIWYFNDQYDK
           *:  : ** * *     :*          :    :*:  :*::* : : :   :

Bz-cmp      QTVWFPTS-SSQDYKTIEPAETTKRFWRPVFIDSSEDKLLASIFDDTSVGNWTVRTDLTA
Es-cmp      QTVFYQTDYFKKDYAKLLQSETSKRHWRPVFIDLSEDLLPTPIFDDTSVGQWVARTDLSA
Fvan-cmp    TTIQYSTPSFTVNFASTFEAETTKRFWRPSRLFLSEDHIITPSFADTSVGKWSHAVDLTK
Gm-CMP      YTAWYDQS--YSGYQTTFNAETTKPFWRPSYLSVSEDHLISSAFVDTDVGSWVARHAMTA
           * :       :     :**:*  ***  :  *** : :  * ** ** *     ::

Bz-cmp      SQLQSEITAQKANNMHPIHISGAGSTET-QYVVIFTEQATPLERNWHAVGDITGFPDNAR
Es-cmp      SELEAEIAAQKAKNLYAVHIAGAGSKGS-KYAVLFAEHLSPLERKWTVTGEVTGFKTNDV
Fvan-cmp    AELKEKIETERAKGLYPIDIQGGGSGSSERFTVVFAERTSPKPRQWNVRGEITGFEDNKA
Gm-CMP      SDLQAEYQTQLSAGRYIIHLQGGGTGANANYAALWATQDIPTPRTFRATGSITGFQNNQA
           ::*: :  :: :  : : : * *:    :  ::: :  *  * :   * :*** *

Bz-cmp      VKRDLDEILQKFMKRNSVRQAQVSASINGTVIASRAYTWAESNRAIVEPSDKFLLGSVSK
Es-cmp      VAKDMDAVMEEFMKKNSVRQAQVAASVNGTVVAERSYTWAESDRAVVKPTDKFGLGSVSK
Fvan-cmp    AEEEVDSIMRRFMEKNGVRQAQFAVALEGKTIAERSYTWAEDDRAIVEPDDIFLLASVSK
Gm-CMP      ASAQADALMQAWMKTNGVRQAQFTVGKNGNILLQKGYSWSEATRHTTAPNDVFLLASNSK
             : * ::  :*: * ***** :  :*  :  : *:*:* *    * * * * * * **

Bz-cmp      AFTYAAVDHLVSTGVVNLTDHIYPLLGYTKPADPRSLNITIQHLLDHTGGFDRGMS-PDI
Es-cmp      MFTYAATTNLLNEGLLNHTTRVYPFLGMNNPADNRSLDITVDHLLQHTAGYNRDIK-PDI
Fvan-cmp    MFLHASIDWLVSHDMLNFSTPVYDLLGY-KPADSRANDINVQHLLDHSAGYDRSMS-GDP
Gm-CMP      MFVEAAVQTLYNSNKLTAATKVYPLLGYTKTTDPRLQQITVDQLLTHYGGLNDTASGFDP
           *  *:  *     :  :  :* :**  : :* *  :* :::** *  * :     *

Bz-cmp      GFIFTTVAQSL--NQSTPATLRQLIEYVLAKPLDFEPGTRSVYSNYGTLLLGYLIANKTG
Es-cmp      GFIFRNIALER--NQTTPVSLRELIEYVYEQPLDFTPGTDSVYSNYGTMLLSYLIANITG
Fvan-cmp    SFMFREIAQSLPTKGAKAATLRDVIEYVVAKPLDFTPGDYSAYSNYCPMLLSYVVTNITG
Gm-CMP      TYRMRDIAIAQG-TGASPASVKNIVDYMSRYTLDYNPGGGYAYSNYGYLLLSYVVEHVTG
           : :  :*     :  :::::::*:   **: **   ****  :** *:: : **

Bz-cmp      ETYMSYLEKNVLK--GLDVEMYTTPGADHTNDRIVQETKFTSISALTPLSEKRVANVYGG
Es-cmp      ESFNSYIHKNVLN--GLDVELYPTSPELNANNPIVQETKYTFYPAQDPASTKQVSNANGG
Fvan-cmp    VPYLDFLEKNILD--GLNVRLYETAASKHTEDRIVQESKNTGQDPVHPQSAKLVPGPHGG
Gm-CMP      MAYYDYLSSALLTPGGYDVRKWVTSPSAHASDPITQESAYTGLNAALPQSQTQVAAVFGG
            : ::  :*  * :*  : *   :: : * **: *    * *  *   **

Bz-cmp      DGAVKEAAVSSFALKASADTVSRFIGKHAAYGLGPRQLFM-----YRDGTVAGARALAYS
Es-cmp      DGSIREEAIGAFGLRASASTISQFLANHAAYDIGPRQAYT-----YRDGTIVGSRAFAQS
Fvan-cmp    DGAVKEECAGTFAMAASASSLAKFIGSHAVWGTGGR-VSS-----NRDGSLSGARAYVES
Gm-CMP      DGMYKEDAFGPASLAASATTLANFIHTHAVWGIGPRPTGTGQWTWARSGSTPGTRTWAQS
           **  :*     : *** ::: *:  ** :  * *       * *:  *:*:  *

Bz-cmp      MA-KLDWAITLNTREYLDENA-----WNTLVFTDLYQLWGALDSALPL
Es-cmp      QD-LIDWSLILNTREYESEQK-----WEQLVFGPISQWY---KYALAE
Fvan-cmp    RG-TIDWALTLNTREYISETE-----FDELRWYSLPDFLS--AFPIAG
Gm_CMP      QWNGLDWAVTVNTRDFPPSNSGQDPFDDVLCESTLPGFLN--AYPTV-
           :**:: :***::          : *   :
```

Figure S1 Multiple sequence alignment. The mature protease sequences were compared by multiple sequence alignment. Conserved amino acids are in grey. Stars represent conserved amino acids; colons represent similar amino acids. The two catalytic motifs are indicated with black boxes.

A

EFLPNQRRSNVTSHVETYYSVDGATHAEKSKALKADGYRIVSLSSYGSPDSANYAAIWVQEEGPSFE
IIHDADEATYNSWLQTWKSRGYVSTQVSATGPAENAVFAGVMENINVANWFQSCELENPWAFSNT
TGNVDVVVKGFRMFGTPEERRYCILGHENVGNEQTTIQYSTPSFTVNFASTFEAETTKRFWRPSRLF
LSEDHIITPSFADTSVGKWSHAVDLTKAELKEKIETERAKGLYPIDIQGGGSGSSERFTVVFA**ERTSP**
KPRQWNVRGEITGFEDNKAAEEEVDSIMRRFMEKNGVRQAQFAVALEGKTIAERSYTWAEDDRAI
VEPDDIFLLASVSKMFLHASIDWLVSHDMLNFSTPVYDLLGYKPADSRANDINVQHLLDHSAGYDR
SMSGDPSFMFREIAQSLPTKGAKAATLRDVIEYVVAKPLDFTPGDYSAYSNYCPMLLSYVVTNITGV
PYLDFLEKNILDGLNVRLYETAASKHTEDRIVQESKNTGQDPVHPQSAKLVPGPHGGDGAVKEECA
GTFAMAASASSLAKFIGSHAVWGTGGRVSSNRDGSLSGARAYVESRGTIDWALTLNTREYISETEFD
ELRWYSLPDFLSAFPIAG

B

EFLPNQRRSNV$_{15}$TSHVETYYSVDGATHAEKSKALKADGYRIVSLSSYGSPDSANYAAIWVQ$_{60}$EEGP
SF$_{66}$EIIHDADEATYNSWLQTWKSRGYVSTQVSATGPAENAVFAGVME$_{110}$NINVAN$_{116}$WFQSCELEN
PWAFSNTTGNVDVVVKGFRMFGTPEERRYCILGHE$_{160}$NVGNEQT$_{167}$TIQYSTPSFTVNFASTFEAETT
KRFWRPSRLFLSEDHIITPSFA$_{211}$DTSVGKW$_{218}$SHAVDLTKAELKEKIETERAKGLYPIDIQGGGSGSS
ERFTVVFA$_{262}$ERTSPKPRQWNVRGEITGFEDNKAAEEEVDSIMRRFMEKNGVRQAQFAVALEGKTI
AERSYTWAEDDRAIVEPDDIFLLASVSKMFLHASIDWLVSHDMLNFSTPVYDLLGYKPADSRANDIN
VQHLLDHSAGYDRSMSGDPSFMFREIAQSLPTKGAKAATLRDVIEYVVAKPLDFTPGDYSAYSNYC
PMLLSYVVTNITGVPYLDFLEKNILDGLNVRLYETAASKHTEDRIVQESKNGQDPVHPQSAKLVPGP
HGGDGAVKEECAGTFAMAASASSLAKFIGSHAVWGTGGRVSSNRDGSLSGARAYVESRGTIDWAL
TLNTREYISETEFDELRWYSLPDFLSAFPIAG

Figure S2 (A) The full-length sequence of Fvan-cmp protease. The N-domain spans residues 1-282, C-domain spans 271-616 with the linker region spanning residues 263-270. The linker region is bolded for visualization within the sequence. (B)The N-domain consists of five structural repeats connected by a small loop. The EHEE repeats are indicated by colour VAL$_{15}$ - GLN$_{60}$ (1), PHE$_{66}$ - GLU$_{110}$ (2), ASN$_{116}$ -GLU$_{160}$ (3), THR$_{167}$ - ALA$_{211}$ (4) and TRP$_{218}$ - ALA$_{262}$ (5).

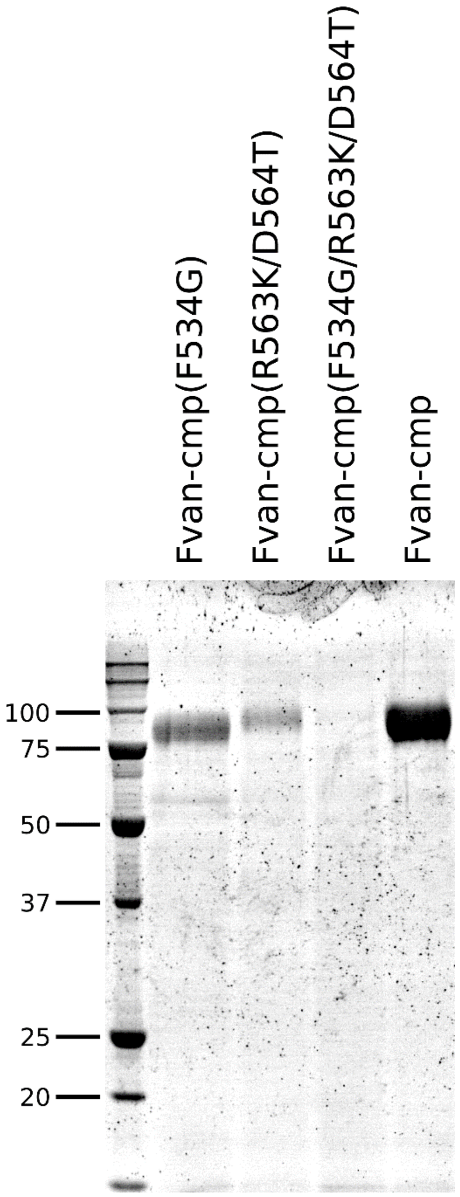

Figure S3 SDS-PAGE and Coomassie staining analysis of cell-free media. After two days of expression, cell-free media from *K. phaffii* cultures expressing either Fvan-cmp or the single, double, or triple mutants was analyzed. The site-directed mutants all showed reduced amounts of protein, likely due to decreased stability of the proteins.

Table S1 Top 50 FoldSeek hits against the AlphaFold Uniprot Database

AlphaFold Entry	Percent Identity	FoldSeek Score	E-value	Organism	Function
AF-A0A166M196	59.1	1480	1.83E-32	*Colletotrichum incanum*	Penicillin-binding protein
AF-A0A135SQZ1	54.6	1462	4.87E-32	*Colletotrichum salicis*	Pectate lyase
AF-A0A1B7YR40	55.3	1438	1.80E-31	*Colletotrichum higginsianum IMI 349063*	Penicillin-binding protein
AF-N4V2J0	56.1	1400	1.42E-30	*Colletotrichum orbiculare MAFF 240422*	Beta-lactamase containing domain
AF-A0A2V1DDC1	53.7	1361	1.18E-29	*Periconia macrospinosa*	Beta-lactamase/transpeptidase-like protein
AF-A0A4R8RCR4	48.6	1230	1.45E-26	*Colletotrichum trifolii*	flp gene product
AF-A0A6A5RWU7	44.9	1227	1.71E-26	*Didymella exigua CBS 183.55*	Beta-lactamase/transpeptidase-like protein
AF-A0A3M7MH02	43.3	1197	8.73E-26	*Pyrenophora seminiperda CCB06*	Penicillin-binding protein
AF-A0A4Q6A4M9	29.8	1092	2.62E-23	*Sphingobacteriales bacterium*	Class A beta-lactamase related serine hydrolase
AF-A0A067T494	36.7	1086	3.63E-23	*Galerina marginata CBS 339.88*	Uncharacterized
AF-A0A1J9QVD6	37.7	1081	4.77E-23	*Diplodia corticola*	Beta-lactamase containing domain
AF-A0A321LBL7	33.4	1067	1.02E-22	*Blastocatellia bacterium AA13*	Beta-lactamase containing domain
AF-A0A6A6B650	35.6	1067	1.02E-22	*Aplosporella prunicola CBS 121167*	Beta-lactamase containing domain
AF-K3V3V4	33.6	1065	1.14E-22	*Fusarium pseudograminearum CS3096*	Beta-lactamase containing domain
AF-A0A409Y2P4	37.7	1044	3.56E-22	*Gymnopilus dilepis*	Beta-lactamase containing domain
AF-A0A067T7P9	30.9	1026	9.46E-22	*Galerina marginata CBS 339.88*	Uncharacterized
AF-A0A1L7TAS3	41.9	972	1.78E-20	*Fusarium mangiferae*	Beta-lactamase containing domain

AlphaFold Entry	Percent Identity	FoldSeek Score	E-value	Organism	Function
AF-A0A3M9ZDX4	27.1	965	2.60E-20	*Leptolyngbya sp. IPPAS B-1204*	Class A beta-lactamase related serine hydrolase
AF-A0A849TPW4	23.7	926	2.17E-19	*Nitrospira sp.*	Uncharacterized
AF-A0A7W7CQE2	29.8	925	2.29E-19	*Actinoplanes abujensis*	Uncharacterized
AF-A0A532CUY9	24.9	912	4.64E-19	*Nitrospira sp.*	Uncharacterized
AF-A0A7W1TEK5	29.3	910	5.17E-19	*Planctomycetes bacterium*	Serine hydrolase
AF-A0A7W0KYF8	29.4	905	6.78E-19	*Acidimicrobiia bacterium*	Serine hydrolase
AF-A0A838DV87	25.6	877	3.10E-18	*Ktedonobacteraceae bacterium*	Serine hydrolase
AF-A0A6H9YRX4	24.6	873	3.86E-18	*Actinomadura rudentiformis*	Uncharacterized
AF-A0A7G5IK55	22.1	872	4.07E-18	*Sandaracinobacter sp. M6*	Serine hydrolase
AF-A0A5J6P455	25.8	869	4.80E-18	*Cellvibrio sp. KY-GH-1*	Class A beta-lactamase related serine hydrolase
AF-A0A3N1JPB1	23.1	843	1.97E-17	*Granulicella sp. GAS466*	Beta-lactamase
AF-A0A2V8HSC7	25.6	823	5.84E-17	*Acidobacteria bacterium*	Beta-lactamase containing domain
AF-A0A3A4B505	25.6	794	2.82E-16	*Bailinhaonella thermotolerans*	Non-specific serine/threonine protein kinase
AF-A0A2J6QYV7	18.9	784	4.86E-16	*Hyaloscypha variabilis F*	Beta-lactamase containing domain
AF-A0A2L2U0F8	29	752	2.76E-15	*Fusarium venenatum*	Beta-lactamase containing domain
AF-D2B7Z4	19.7	746	3.83E-15	*Streptosporangium roseum DSM 43021*	Beta-lactamase
AF-A0A3N7JU83	21.1	743	4.51E-15	*Albitalea terrae*	Class A beta-lactamase related serine hydrolase
AF-A0A7Y6IQ54	22.1	733	7.76E-15	*Nonomuraea rhodomycinica*	Uncharacterized
AF-A0A1H1BZ32	22.5	724	1.27E-14	*Thermostaphylospora chromogena*	Uncharacterized
AF-A0A7X0U1N1	17.8	723	1.34E-14	*Nonomuraea rubra*	Uncharacterized
AF-A0A2J6SIY4	33.3	719	1.66E-14	*Hyaloscypha bicolor E*	Beta-lactamase/transpeptidase-like protein

AlphaFold Entry	Percent Identity	FoldSeek Score	E-value	Organism	Function
AF-A0A367FK14	20.8	716	1.96E-14	*Sphaerisporangium album*	Uncharacterized
AF-A0A7W8ECQ3	22.5	696	5.80E-14	*Nonomuraea endophytica*	Non-specific serine/threonine protein kinase
AF-A0A5R8MSZ6	18.8	695	6.12E-14	*Nonomuraea sp. KC401*	Uncharacterized
AF-A0A5S4FIC2	20.1	694	6.46E-14	*Nonomuraea turkmeniaca*	Uncharacterized
AF-A0A848DM22	15	691	7.60E-14	*Pseudonocardia bannensis*	Beta-lactamase containing domain
AF-A0A0J9ECQ5	19.3	683	1.17E-13	*Candidatus Rhodobacter lobularis*	Beta-lactamase containing domain
AF-A0A553Y292	16.5	676	1.72E-13	*Streptomyces benahoarensis*	Beta-lactamase containing domain
AF-A0A2H3RIQ8	35.4	672	2.14E-13	*Fusarium fujikuroi*	Beta-lactamase containing domain
AF-A0A124DZT4	22.9	659	4.33E-13	*Mycolicibacterium brisbanense*	Beta-lactamase containing domain
AF-A0A239BKE8	20.3	646	8.77E-13	*Streptosporangium subroseum*	Uncharacterized
AF-A0A4Q5NQF3	19.2	626	2.60E-12	*bacterium*	Beta-lactamase containing domain
AF-A0A022VXE6	14	624	2.90E-12	*Trichophyton rubrum CBS 288.86*	Uncharacterized

The matches are sorted based on their FoldSeek score metric. All proteins that matched the Fvan-cmp N-domain, none characterized the purpose of the tertiary fold. Within the top 50 results, 20 proteins were from the fungal domain and 30 proteins were from the bacterial domain.

Table S2 Conserved β-lactamase shell residues

Residue	Fvan-cmp residue*	Shell	Function
E37	Q308	III	Proper folding
(A, D, Q, S)			
R65	P335	III	Loop interactions
(A, T, P, L, H, K, C)			
T71	L341	II	Reduces mobility of active site serine
(S, A, V, L)			
D131	D403	II	Stability and global positioning
A185	S456	III	
(S, T, V, E, Q, R, N, G)			
W229	S501	III	Hydrophobic and stacking interactions
(S, A, Y, C, F)			

*The Fvan-cmp C-domain begins at residue 271, so the corresponding residue was scaled up to fit the full sequence length.

The residues conserved across Class A β-lactamases and are designated to shells based on proximity to the active site and their related function. The bracketed residues are the conserved alternates found in nature. Conserved residues shared between polyglycine hydrolases and β-lactamases are listed.
